# Lactulose in cirrhosis: Current understanding of efficacy, mechanism, and practical considerations

**DOI:** 10.1097/HC9.0000000000000295

**Published:** 2023-10-12

**Authors:** Patricia P. Bloom, Elliot B. Tapper

**Affiliations:** Department of Internal Medicine, Division of Gastroenterology and Hepatology, University of Michigan, Ann Arbor, Michigan, USA

## Abstract

HE is a complication of cirrhosis characterized by neuropsychiatric and motor dysfunction, and results in decreased quality of life and increased mortality. Lactulose is a synthetic disaccharide used to treat HE since 1966, though many questions about its use remain unanswered. Lactulose reverses minimal HE, prevents overt HE, improves quality of life, increases the rate of recovery from overt HE, and improves survival rates. Lactulose’s clinical effect appears to be derived from its impact on intestinal microbes, likely a result of its enteric acidifying effect, positive pressure on beneficial taxa, and improvement of gut barrier function. There are several practical considerations with lactulose including (1) a need to avoid excessive bowel movements and subsequent dehydration, (2) treatment titration protocols need further investigation, (3) baseline or treatment-induced gastrointestinal side effects limit adherence in some cases, and (4) the utility of monitoring stool consistency or pH remains unknown. Further research is needed to optimize our use of this effective treatment for HE.

## INTRODUCTION

HE is a complication of cirrhosis characterized by neuropsychiatric and motor dysfunction. Manifestations can range from subtle (minimal HE) to severe (overt HE) and even coma. HE is associated with considerable patient and caregiver burden, decreased quality of life, and poor survival.^1–3^ Lactulose has been used to treat HE since 1966, yet our understanding of how to best use lactulose in clinical treatment plans and its mechanism remains relatively unclear.

Lactulose is a synthetic disaccharide composed of 1 molecule of galactose and 1 molecule of fructose.^4^ Only 0.3% of lactulose is absorbed by the gastrointestinal tract in healthy adults, so lactulose makes its impact within the intestinal lumen.^5^ Lactulose’s clinical effect appears to be derived from its impact on intestinal microbes, likely a result of its pH-lowering effect, positive pressure on beneficial taxa, and improvement of gut barrier function.

## CLINICAL EFFICACY OF LACTULOSE TO TREAT HEPATIC HE

**FIGURE 1:**
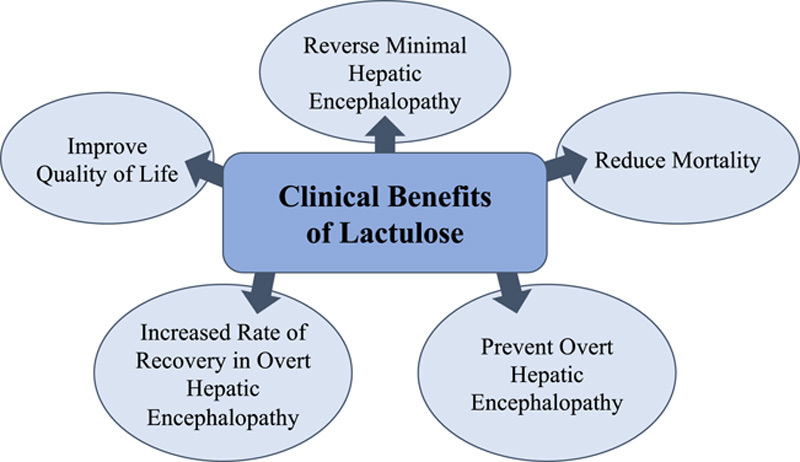
Clinical benefits of lactulose. Lactulose has multiple clinical benefits for patients with cirrhosis and HE.

Lactulose is an effective treatment for HE (Figure 1). A Cochrane review including 38 randomized controlled trials of nonabsorbable disaccharides found a beneficial effect on HE (RR=0.58, 95% CI: 0.5–0.69).^6^ Lactulose also reduced mortality to 8.5% in patients with cirrhosis and overt HE versus 14% among those with overt HE not taking lactulose.^6^ A network meta-analysis of 25 trials found that when comparing lactulose, rifaximin, probiotics, and l-ornithine l-aspartate (an ammonia-lowering agent) for the treatment of minimal HE, lactulose was the only agent able to meet all 3 endpoints: reverse minimal HE, prevent overt HE, and improve quality of life.^7^ Although many patients experience breakthrough overt HE episodes while on lactulose,^8^ lactulose withdrawal markedly increases the risk of breakthrough in patients on rifaximin.^9^

### Lactulose for overt HE

Lactulose is the first-line therapy for overt HE.^10^ Despite this recommendation, high-quality data are relatively limited. Specifically, there are no controlled multicenter trials demonstrating lactulose efficacy for overt HE treatment or primary prevention. In the original controlled studies of lactulose compared with sorbitol, it was effective at inducing resolution of altered mental status and particularly after cross-over to lactulose after sorbitol-induced worsened cognitive function.^11,12^ Compared with a glucose control, lactulose also increased the rate of mental status recovery in overt HE.^13^ In a randomized trial, lactulose delivered by means of enema compared with tap water enemas was stopped early for efficacy.^14^ Frequent lactulose doses until improving mental status has been associated with improved hospital length of stay in nonrandomized trials.^15,16^ When combined with or when followed by large-volume polyethylene glycol (PEG) colonic purges, the time to mental status normalization is shortened.^17,18^ Lactulose is also highly effective for the secondary prevention of overt HE episodes.^19^ Breakthrough overt HE during lactulose therapy is most often related to nonadherence (ie, consuming no or insufficient lactulose), but closely followed by dehydration from excessive bowel movements.^20^

### Lactulose for minimal HE

Lactulose therapy is associated with consistent improvements in health-related quality of life. This has been observed using validated indices such as the Sickness Impact Profile,^21,22^ Euro-QOL,^23^ and the Modified Chinese Quality of Life Questionnaire.^24^ The impact is particularly clear for subscales involved in social activities, home management, emotional behavior, and sleep functioning.^21,22^ In a randomized trial of crystalline lactulose for patients enrolled on the basis of high activity impairment, 28 days of lactulose did not significantly improve health-related quality of life measured using the Short Form-8 but did improve activity impairment, sleep quality, and cognitive function measured using the Animal Naming Test.^25^

## THERAPEUTIC MECHANISM OF LACTULOSE

### Lactulose metabolism in the gut

Lactulose is a synthetic disaccharide composed of one molecule of galactose and one molecule of fructose.^4^ Only 0.3% of lactulose is absorbed by the gastrointestinal tract in healthy adults, so lactulose makes its impact within the intestinal lumen.^5^ Lactulose is metabolized by bacterial enzymes into carbon dioxide and short-chain fatty acids (SCFAs).^4^ Lactulose increases fecal nitrogen excretion in rats with gut bacteria, but not in germ-free rats, supporting the role of bacteria in lactulose metabolism.^26^

### Lactulose absorption is minimal

In 2021, it was shown in multiple experiments involving healthy controls and patients with diarrhea-type irritable bowel syndrome that <0.4% of lactulose is absorbed using radiolabeled ingestions.^5^ The majority of that absorption occurs in the small bowel, based on the finding that most urinary lactulose is collected within 8 hours of consumption. There was no effect of age, sex, or the presence of irritable bowel syndrome on lactulose absorption. Another study performed a multisugar test (designed to evaluate intestinal permeability) in patients with compensated cirrhosis and controls.^27^ While they did not report the absorption of lactulose alone, there was no difference in the absorbed ratio of lactulose/rhamnose between patients with cirrhosis and controls. Likely very little lactulose is absorbed by the intestine, unmetabolized, in patients with cirrhosis.

### Location of lactulose metabolism

The geography of lactulose fermentation is challenging to ascertain, because it is technically difficult to access the distal small bowel and proximal colon. Despite this challenge, several studies suggest that lactulose metabolism occurs in the colon.

In a 1965 study, Dahlqvist and Gryboski^28^ took sections of human small intestine, obtained from surgical resections, and incubated them with lactulose *in vitro*. The small intestine sections were able to hydrolyze lactose, but not lactulose. It is not clear if small intestinal bacteria were included in this experiment, but the authors conclude that lactulose is not hydrolyzed by human small intestine disaccharidases. This study does not address the possibility that small intestinal bacteria may metabolize lactulose, which to our knowledge has never been studied.

The microbial breakdown of carbohydrates is fermentation, which yields SCFA as the main end product. As such, SCFA can be used as a marker of the location of lactulose fermentation. One study performed an autopsy of 6 adult humans within 4 hours of death and found the following mean total SCFA levels: jejunum (<1 mmol/kg), ileum (13 mmol/kg), cecum (131 mmol/kg), ascending colon (123 mmol/kg), transverse colon (117 mmol/kg), and descending colon (80 mmol/kg).^29^ While these autopsy patients were not consuming lactulose, this work suggests that the proximal colon is where most carbohydrate fermentation and therefore lactulose metabolism occurs.

Another study administered lactulose 20 g twice daily to healthy volunteers for 8 days.^30^ Four healthy volunteers underwent colonoscopic collection of ileal and colonic contents on days 1 and 8 of radiolabeled (^14^C) lactulose. There was an increase in ^14^CO_2_ in breath testing over the 8 days, suggesting that carbon from lactulose breakdown products is absorbed into systemic circulation. Over 8 days, ileal fluid did not change in pH or SCFA concentrations. However, the cecum had a significant drop in pH and increase in lactate, acetate, and total volatile fatty acid concentrations. Fecal pH and fatty acid concentrations did not change. Therefore, it appears that the bulk of lactulose metabolism in a healthy individual is occurring in the cecum, and colonic SCFAs are used before they are excreted in stool.

Finally, it is clear that lactulose can be metabolized by colonic bacteria. One group anaerobically cultured 64 colonic bacterial strains in lactulose-containing media.^31^ They found that some organisms were not able to metabolize lactulose, but many were. Lactulose metabolizers *in vitro* included *Bacteroides*, *Bifidobacterium*, *Clostridia*, and *Lactobacilli*.

### Lactulose as a prebiotic

Prebiotics are substrates selectively used by host microorganisms that confer a health benefit.^32^ Prebiotics are most often nonabsorbable carbohydrates, able to be fermented by gastrointestinal bacteria, leading to an increased abundance of beneficial taxa and SCFA production.

Clinical reports of lactulose started in 1957 when Petuely^33^ reported that lactulose increased fecal *Lactobacillus bifidus* when fed to adults and infants. Early culture-based studies demonstrated that lactulose promotes the growth of *Lactobacillus* and *Bifidobacteria*, both microorganisms that confer a health benefit, and the production of SCFAs butyrate, acetate, and propionate.^34–40^ Elkington et al^11^ performed the first double-blind clinical trial of lactulose to treat HE, and found that lactulose consistently resulted in the presence of lactobacilli in the stool. The glycoside hydrolase able to hydrolyze lactulose was found in nearly all of the 144 *Bifidobacteria* strains available in the National Center for Biotechnology Information in 2021, again highlighting the strong relationship between lactulose and this bacteria.^41^ Based on cecal lactulose concentrations, pH, and rate of SCFA production, it is likely that bacterial ability to metabolize lactulose increases over 8 days.^30^ While early culture-based studies reproducibly showed an increase in *Lactobacillus* and *Bifidobacteria* with lactulose, it remained unclear if the prebiotic effect of lactulose and subsequent changes in bacterial abundance influence clinical outcomes in patients with cirrhosis and HE. For example, lactulose can often resolve overt HE within 1–2 days; however, older data suggest that increases in probiotic bacteria are not observed for ≥2 days.^42^ However, these studies are very small, confounded, and used culture-based techniques, sometimes analyzing stool that had been at room air temperature without DNA preservative for 24 hours.^12^

A minority (<30%) of gut bacteria can be cultured. Therefore, culture-based methods limit our ability to evaluate microbiota changes with lactulose.^43^ Since the 1990s, culture-independent techniques such as analysis of 16S rRNA amplicon sequencing have increased the breadth and taxonomic depth of microbiota analysis. Analyzing 16S rRNA amplicons from stool, a multicenter trial of lactulose in 98 patients with minimal HE found an increase in 3 bacterial families. Furthermore, they found that lactulose-induced fecal microbial abundance changes differed between patients with and without clinical response.^24^ Of note, this study reported family-level taxa (not the more granular genus level), and did not report a change in *Lactobacillus* and *Bifidobacteria*. Another group studied paired stool specimens in 21 patients with cirrhosis prelactulose and postlactulose for 6 weeks.^44^ Investigating bacteria found in at least 10% of samples using 16S rRNA amplicon sequencing, they found no taxa whose abundance differed significantly in a paired *t* test (and controlling for multiple comparisons). They found that interpatient differences in microbiota community structure were greater than intrapatient changes with lactulose. However, the principal coordinate analysis showed that individual’s microbial community structure did indeed change with lactulose. This result suggests that microbial communities change with lactulose, but may do so in a heterogeneous way. In a study of 7 men in whom lactulose was withdrawn, an amplicon-based technique similar to 16S rRNA amplicon sequencing showed that fecal *Faecalibacterium* abundance dropped from 6% to 1% with lactulose withdrawal.^45^ Finally, while none of the amplicon-based sequencing studies in patients with cirrhosis found an increase in *Lactobacillus* and *Bifidobacteria* with lactulose as had been seen in culture-based studies, a healthy human model found that lactulose promotes re-growth of *Bifidobacterium* (with amplicon sequencing methods) and butyrate production after antibiotic administration.^46^

Techniques for microbial community analysis continue to evolve beyond amplicon-based sequencing.^47^ Metagenome approaches have several advantages over amplicon-based techniques like 16S rRNA sequencing, including greater taxonomic resolution and ability to infer bacterial function. To our knowledge, no one has investigated microbial community composition and functional changes with lactulose using these modern metagenome analysis techniques.

### Lactulose as it relates to ammonia

In the 1970s, a series of experiments performed in a fecal incubation system characterized some fundamentals about the relationship between lactulose, enteric bacteria, and ammonia. First, Vince et al^48^ found that lower fecal pH led to less bacterial ammonia production. This is relevant because lactulose leads to SCFA production and stool acidification, so this acidification may diminish bacterial ammonia production. Second, administering lactulose significantly decreased ammonia production from most bacteria.^49^ Third, even when buffering for a consistent pH, lactulose reduced enteric bacteria ammonia production.^50^ The authors concluded that the majority of ammonia-lowering effect was attributed to the direct lactulose effect on bacterial fermentation, and less so from pH lowering. Lactulose fermentation by probiotic taxa requires increased bacterial amino acid synthesis using ammonia as the substrate, leading to reduced luminal ammonia concentrations.^50–53^

Lactulose may reduce serum ammonia through additional mechanisms, including trapping ammonium ions in the colon.^54–56^ Elkington et al^11^ performed the first double-blind clinical trial of lactulose to treat HE, and found that lactulose lowered arterial ammonia levels and stool pH in 5 of 7 patients. Here, as elsewhere, lactulose both improved the patient’s mental processing and lowered ammonia levels; however, ammonia levels rarely normalize.^23^ The distribution of ammonia between colonic contents and colonic venous blood relates to colonic pH. Above a fecal pH of 6.2, ammonia fluxes from the colonic lumen to blood. However, below the fecal pH of 6.2, ammonia flux reverses and travels from the blood to the colonic lumen.^4,12^ At low fecal pH, the theory is that ammonia diffuses from the blood into the gastrointestinal lumen, is converted to ammonium ions, and is disposed of through feces. Work in healthy humans supports this possibility, as lactulose leads to an increase in total ammonia (and nitrogen) excretion.^57^

### Lactulose as a laxative agent

The first double-blind clinical trial of lactulose to treat HE found that lactulose was more clinically effective than sorbitol despite both inducing loose stool.^11^ However, only 1 patient had active HE symptoms at the start of the trial and despite resolution after cross-over from sorbitol to lactulose, conclusions from this study are uncertain. Magnesium sulfate was also shown to produce diarrhea without clinical improvement in HE.^58^ Therefore, while lactulose produces a laxative effect, it is not clear that this correlates strongly with clinical improvement.

### Lactulose and potential pathogens

Fermentation of lactulose leads to an increased abundance of beneficial taxa that can use these substrates, produce SCFAs, and reduce pH in the intestinal lumen. Increased biomass of beneficial taxa reduces available nutrients for invading microbial pathogens.^59^ In the setting of colonic acidification from SCFAs, ammonia production from gram-negative bacteria decreases, likely reflecting diminished metabolic activity as well as growth inhibition of those bacteria.^50^ A recent multicenter study of lactulose for minimal HE found no significant change in microbial composition (using 16s rRNA sequencing); however, those with a clinical response experienced a significant decrease in certain Actinobacteria, Bacteroidetes, Firmicutes, and Proteobacteria relative to nonresponders.^24^ Bacteroidetes and Proteobacteria produce lipopolysaccharide, which has been implicated in the pathogenesis of HE.^60–62^

### Lactulose and barrier function

*In vitro* and human studies showed that prebiotics such as galactooligosaccharides improve intestinal barrier function by stimulating mucus-producing goblet cells, augmenting tight junction assembly, and mitigating inflammation.^63–66^ In patients with minimal HE, lactulose decreases bacterial DNA in the serum and improves neurocognitive test scores, presumably through changes to bacterial composition and improved intestinal permeability—the latter of which may be a result of increased SCFA production.^67^

## SUMMARY

**FIGURE 2:**
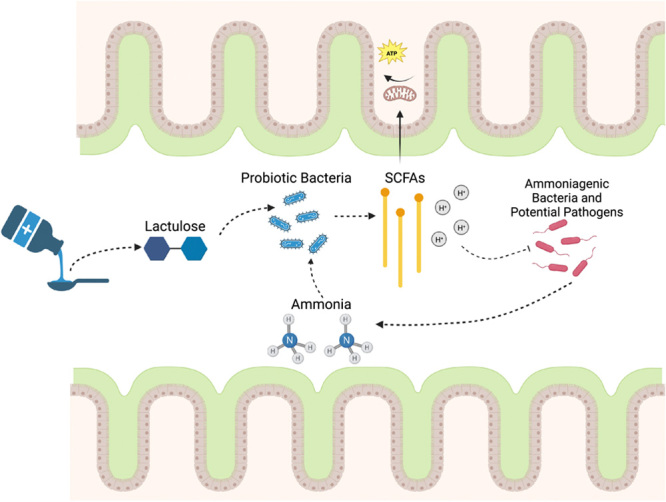
Mechanisms by which lactulose treats HE. Lactulose puts positive pressure on probiotic bacteria, which consume ammonia and produce SCFAs as part of their metabolism. These SCFAs supply energy to the intestinal epithelia and also create an acidic environment which place negative pressure on ammonia-producing and potentially pathogenic organisms. Figure created in biorender.com. Abbreviation: SCFA, short-chain fatty acid.

While the literature is not uniform on its actions, it appears that lactulose’s benefits in HE are mediated through changes in intestinal microbes, likely a result of its pH-lowering effect, positive pressure on beneficial taxa, and an improvement in gut barrier function (Figure 2).

In 1969, Haemmerli and Bircher^42^ wrote “the exciting aspect of lactulose therapy, however, is its mechanism of action. Once completely elucidated, it may shed new light on the pathogenesis of hepatic coma.” Nearly half a century later, we have a better understanding of lactulose’s mechanism of action, but the picture is still not complete.

## PRACTICAL CONSIDERATIONS

### Achieving balance

The oft-repeated target for lactulose titration is to achieve 2–3 soft bowel movements daily. In view of the known mechanisms of lactulose effects, it is clear that its benefits are facilitated in part by, but not exclusive to, catharsis. Increased bowel movement frequency alone is not an effective use of lactulose. Diarrhea, for example, is a common reason for treatment discontinuation and can lead to dehydration or electrolyte loss, which are, themselves, triggers for HE. For those with overt HE, early and effective laxation in the context of euvolemia and supportive care with additional doses is advisable.^15^ However, after the resolution of disorientation, the number of bowel movements is no longer associated with 30-day outcomes.^68^ This finding extends to a cohort of 269 outpatients with ≥6 months of medication adherence, where achieving a target of 2–3 bowel movements daily was not associated with cognitive function on psychometric testing.^69^ In a retrospective study of 112 patients on lactulose, a Bristol Stool Scale >4 was found to be associated with a reduced risk of hospitalization for HE even among patients not meeting the target of 2–3 bowel movements.^70^

The concept of titrating lactulose to qualitative stool changes is attractive. However, many patients with cirrhosis have baseline disturbances in bowel function such as irritable bowel syndrome. Further, there was no association between quality of life and cognitive function and the Bristol Scale in a randomized trial of lactulose.^25^

**FIGURE 3:**
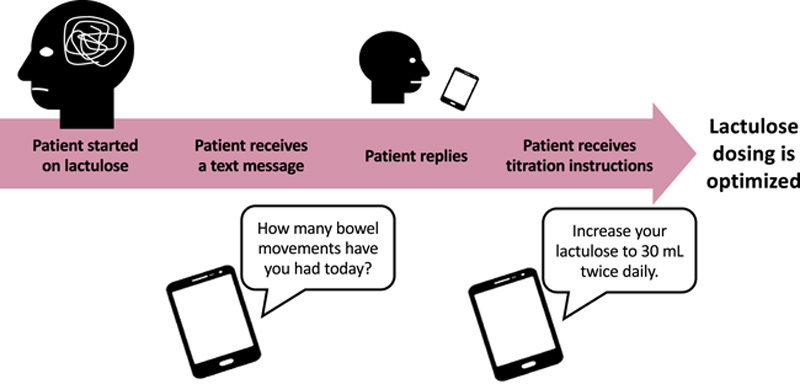
Use of mobile technology to titrate lactulose. Patients taking lactulose require monitoring to avoid excessive bowel movements and subsequent dehydration. Such monitoring can be achieved with just-in-time text messages or other mobile technology.

Moving forward, there are several areas to consider. First, safety is paramount and closer monitoring of patients to avoid excessive bowel movements is important to avoid dehydration. This can be achieved using just-in-time text-message-based instructions on how to adjust doses based on the prior day’s bowel movements as was performed in the MiKristal trial (Figure 3).^25^ Second, the method of treatment initiation and titration has not undergone any scrutiny or investigation. Slowly increasing the amount of lactulose consumed by volume per dose and frequency, titrating to bowel movement frequency and ideally patient-reported outcomes may be advantageous. Third, further validation of qualitative stool changes in longitudinally followed patients is warranted. Targets include the Bristol scale, stool pH, and even microbial composition.

### Alternatives

Given the risk of bloating with and sweetness of lactulose, there is interest in finding alternatives. In practice, though unstudied, many will use PEG. In a randomized trial, Rahimi et al^17^ found that 4 L of PEG hastened mental status recovery compared with lactulose 30 mL thrice daily. However, 87% of PEG subjects received lactulose before randomization, and thus, the comparison is best framed as PEG-plus-lactulose versus low-dose lactulose alone. In a recent small trial, 2 L of PEG followed by lactulose was more effective than low-dose lactulose alone.^18^ Taken together, these studies confirm the importance of early and effective laxation in the management of overt HE while also highlighting the role of lactulose. PEG has never been explored for the prevention of HE. Many patients may never, or rarely, develop recurrent HE after an episode of overt HE, making questionable the anecdotal experiences of success after switching to PEG. As such, it is premature to generalize these inpatient studies to the long-term management of outpatients. Indeed, many prior studies conducted cross-over assessments with inert cathartics such as sorbitol or magnesium with poor outcomes.^11,12^ Trials of PEG are needed in carefully selected patients where equipoise exists before this strategy can be endorsed. Finally, given its tolerability, many patients are started on rifaximin alone. However, patients receiving rifaximin alone are more likely to have recurrent hospitalizations for HE. Relative to “no therapy,” lactulose alone was associated with a lower incidence rate ratio for hospital-days per person-year than those receiving rifaximin alone: 0.31 (95% CI: 0.30–0.32) versus 0.49 (95% CI: 0.45–0.53).^3^

## FUTURE DIRECTIONS

**FIGURE 4:**
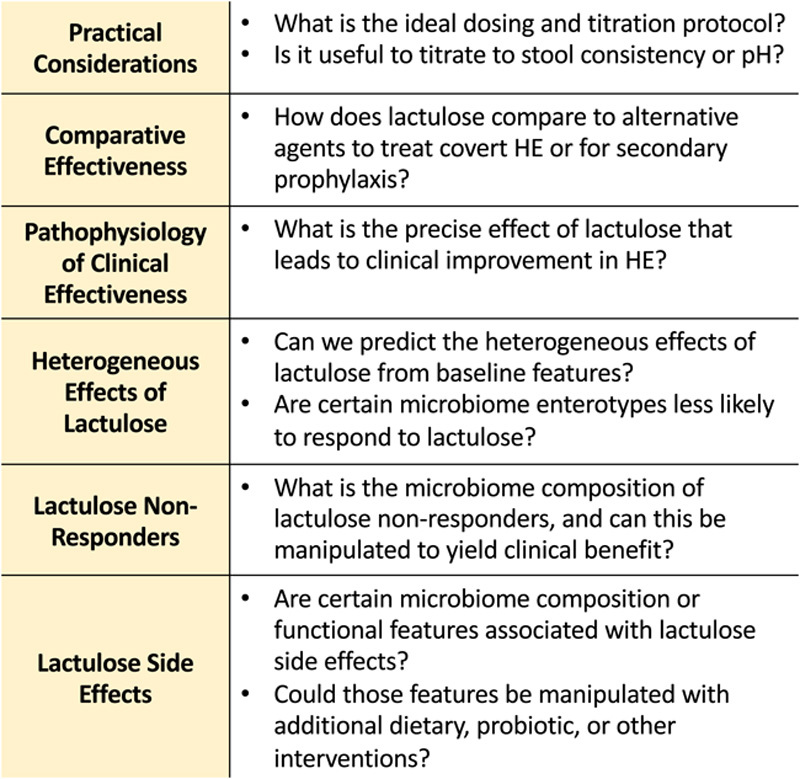
Aims of future lactulose research. While lactulose is clearly effective in treating HE, there are several lingering uncertainties about its mechanism and ideal use. These uncertainties should be the focus of future research.

Lactulose has been used to effectively treat HE for 50 years. However, some uncertainties remain about its mechanism and ideal use. There appears to be heterogeneous effects of lactulose clinically, as well as on the microbiome. Future research should employ modern metagenome analysis techniques to investigate microbial community composition and functional changes with lactulose, including analysis of diverging subgroups (Figure 4). For example, are there certain bacterial enterotypes that are less or more likely to respond to lactulose clinically? What is the microbiome community structure of lactulose nonresponders, and could this be manipulated to lead to clinical success? Finally, gastrointestinal side effects are unfortunately common with lactulose use. Are there certain microbiome composition or functional features associated with lactulose side effects, and could they be manipulated with other dietary, probiotic, or other interventions?
